# Sea anemones (Cnidaria, Anthozoa, Actiniaria) from coral reefs in the southern Gulf of Mexico

**DOI:** 10.3897/zookeys.341.5816

**Published:** 2013-10-08

**Authors:** Ricardo González-Muñoz, Nuno Simões, José Luis Tello-Musi, Estefanía Rodríguez

**Affiliations:** 1 Unidad Multidisciplinaria de Docencia e Investigación en Sisal (UMDI-Sisal), Facultad de Ciencias, Universidad Nacional Autónoma de México (UNAM); Puerto de Abrigo, Sisal, Yucatán, México, C. P. 97356; 2 Posgrado en Ciencias del Mar y Limnología, UNAM; Instituto de Ciencias del Mar y Limnología, Circuito Exterior, Ciudad Universitaria, Ciudad de México, C. P. 04510; 3 Laboratorio de Zoología, Facultad de Estudios Superiores Iztacala (FES-I), UNAM; Avenida de los Barrios 1, Los Reyes Iztacala, Estado de México, C. P. 54090; 4 American Museum of Natural History, Division of Invertebrate Zoology, Central Park West at 79th Street, New York, NY 10024, USA

**Keywords:** Anthozoa, Veracruz Reef System, Cayo Arenas, Alacranes Reef, Banco de Campeche, Yucatán

## Abstract

Seven sea anemone species from coral reefs in the southern Gulf of Mexico are taxonomically diagnosed and images from living specimens including external and internal features, and cnidae are provided. Furthermore, the known distribution ranges from another 10 species are extended. No species records of sea anemones have been previously published in the primary scientific literature for coral reefs in the southern Gulf of Mexico and thus, this study represents the first inventory for the local actiniarian fauna.

## Introduction

**Figure 1.**
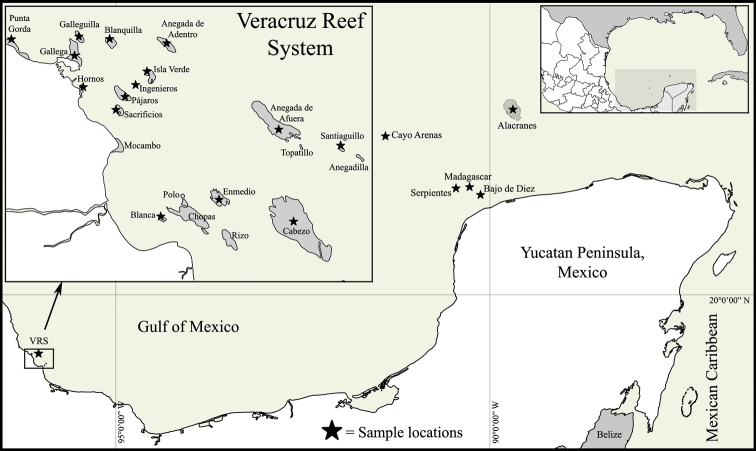
Map of the Southern Gulf of Mexico, indicating the localities sampled in this study.

**Figure 2.**
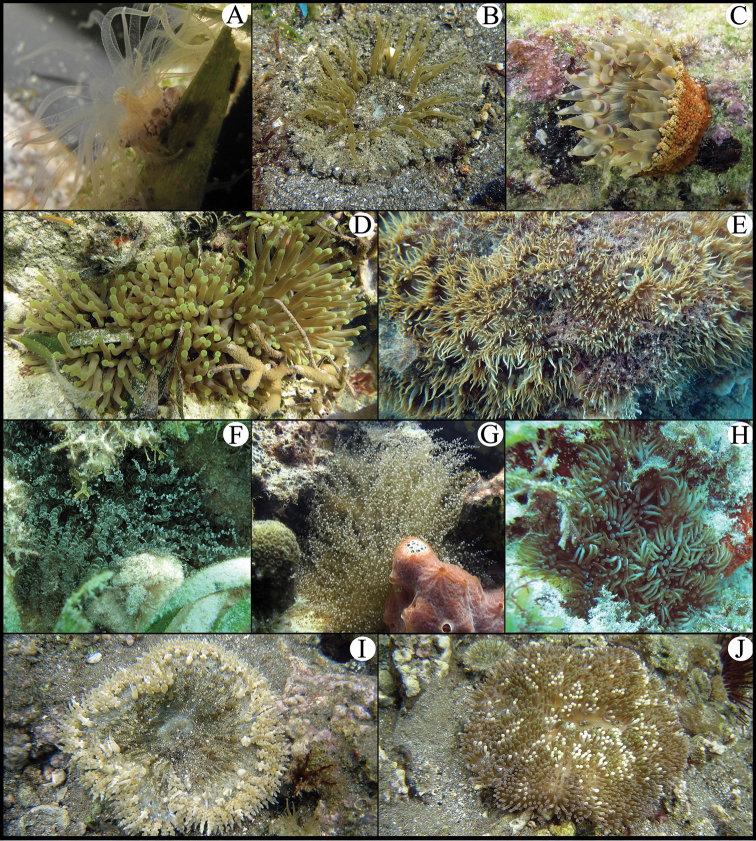
**A** *Bunodeopsis antilliensis* **B** *Actinostella flosculifera* **C** *Bunodosoma granuliferum* **D** *Condylactis gigantea* **E** *Aiptasia pallida* **F** *Bartholomea annulata* **G** *Ragactis lucida* **H** *Lebrunia danae* **I** *Phymanthus crucifer* **J** *Stichodactyla helianthus*.

Sea anemones (order Actiniaria) are among the benthic and sessile invertebrates inhabiting the southern Gulf of Mexico (SGM) coral reefs. Nevertheless, sea anemones are typically overlooked in assessments of coral reefs biodiversity due to the poor taxonomic knowledge available on local species. Although some studies provide records of sea anemone species from some coral reefs in the SGM (González-Solís 1985, Rosado-Matos 1990, González-Muñoz 2005, Vélez-Alavéz 2007, CONANP 2006), formal taxonomic identification was beyond their scope. Thus, no inventory of sea anemones has been previously published in the primary scientific literature for coral reefs in the SGM. The present contribution documents 17 species from 15 coral reefs of the Veracruz Reef System (VRS), and five coral reefs of the Campeche Bank, Yucatán Peninsula (Figure 1). Taxonomic diagnoses with images of living specimens, including external and internal features, and cnidae are provided for seven species: *Anemonia sargassensis* Hargitt, 1908; *Anthopleura pallida* Duchassaing and Michelotti, 1864; *Bunodosoma cavernatum* (Bosc, 1802); *Isoaulactinia stelloides* (McMurrich, 1889); *Actinoporus elegans* Duchassaing, 1850; *Lebrunia coralligens* (Wilson, 1890); and *Calliactis tricolor* (Le Sueur, 1817). The other 10 species were recently diagnosed in an inventory of the Mexican Caribbean sea anemone fauna (González-Muñoz et al. 2012); however, here we extend their distribution range for coral reef localities in the SGM (Table 1). Those species are: *Bunodeopsis antilliensis* Duerden, 1897; *Actinostella flosculifera* (Le Sueur, 1817); *Bunodosoma granuliferum* (Le Sueur, 1817); *Condylactis gigantea* (Weinland, 1860); *Lebrunia danae* (Duchassaing & Michelotti, 1860); *Phymanthus crucifer* (Le Sueur, 1817); *Stichodactyla helianthus* (Ellis, 1768), *Aiptasia pallida* (Agassiz in Verrill, 1864); *Bartholomea annulata* (Le Sueur, 1817); and *Ragactis lucida* (Duchassaing & Michelotti, 1860) (Figure 2). Although these 17 species have a widespread geographic distribution in the Caribbean Sea and Gulf of Mexico (Fautin and Daly 2009, Fautin 2013), this study represents the first inventory of sea anemones of coral reefs in the SGM. The aim of this contribution is to encourage biological and ecological research on sea anemones of the coral reefs of the SGM by facilitating identification work.

**Table 1.** Distribution of sea anemones found on the coral reefs of SGM in the present study. The symbol “x” indicates localities of previous but not published records, “*” indicates new records for the locality found in the present study, and “†” indicates new records for Mexico.

	**Veracruz Reef System**															**Campeche Bank Reefs**				
**Species**	**Punta Gorda**	**Galleguilla**	**Gallega**	**Blanquilla**	**Anegada de Adentro**	**Hornos**	**Isla Verde**	**Pájaros**	**Isla Sacrificios**	**Ingenieros**	**Anegada de Afuera**	**Santiaguillo**	**Blanca**	**Isla de Enmedio**	**Cabezo**	**Bajo de Diez**	**Madagascar**	**Serpientes**	**Alacranes**	**Cayo Arenas**
*Bunodeopsis antilliensis* Duerden, 1897																			*	*
*Actinostella flosculifera* (Le Sueur, 1817)	*	x	*			*	x		*	*				*	*		*		*	
*Anemonia sargassensis* Hargitt, 1908	*		*			*	x			*									*	*
*Anthopleura pallida* Duchassaing & Michelotti, 1864 †																			*	
*Bunodosoma cavernatum* (Bosc, 1802) †	*	*	*			*	*			*				*						
*Bunodosoma granuliferum* (Le Sueur, 1817)														*					*	
*Condylactis gigantea* (Weinland, 1860)																*	*		x	*
*Isoaulactinia stelloides* (McMurrich, 1889) †	*		*						*						*					
*Aiptasia pallida* (Agassiz in Verrill 1864)	*	x	*			*			*	*			*				*	*	*	
*Bartholomea annulata* (Le Sueur, 1817)		*	*				*	*	*		*			*	*	*	*	*	*	*
*Ragactis lucida* (Duchassaing & Michelotti, 1860)		*							*		*								*	
*Lebrunia coralligens* (Wilson, 1890)		x		*	*		x	*	*	*		*	*	*	*					
*Lebrunia danae* (Duchassaing & Michelotti, 1860)																			*	
*Actinoporus elegans* Duchassaing, 1850 †			*				*		*					*	*					
*Calliactis tricolor* (Le Sueur, 1817) †																	*		*	
*Phymanthus crucifer* (Le Sueur, 1817)	*	x	*		*	*	x	*	*	*				*	*		*		*	*
*Stichodactyla helianthus* (Ellis, 1768)	*	x	*	*		*	x		*	*				*	*				x	

## Methods

Observations and collections of specimens were done at 20 coral reef localities of the SGM during 2009–2011 (Figure 1). Habitats sampled include sandy patches, seagrass meadows, rocky pavement, coral rubble, and coral patches in several zones of coral reefs, and depth and habitat characteristics were recorded. Specimens were collected by hand, either by snorkeling or SCUBA diving, using a small shovel, and hammer and chisel. Collected specimens were transferred to the laboratory and maintained in an aquarium to photograph their color in life. Specimens were relaxed using 5% MgSO_4_ seawater solution and subsequently fixed in 10% formalin in seawater. Measurements provided for pedal disc, column, oral disc and tentacles were obtained from living and relaxed specimens. Fragments of selected specimens were dehydrated and embedded in paraffin. Histological sections 6–10 µm thick were stained with hematoxylin-eosin (Estrada et al. 1982) or Ramón and Cajal’s Triple Stain (Gabe 1968). For cnidae examination, squash preparations of small amounts of tissue of two specimens from each species (tentacles, actinopharynx, filaments, column, and if present, marginal projections, acrorhagi, acontia and pseudotentacles) were examined using a Nikon Labophot-2 light microscope (1000x oil immersion), photographed and haphazardly measured. Nematocyst terminology follows Mariscal (1974) and Östman (2000).

Specimens were deposited in the Collection of the Gulf of Mexico and Mexican Caribbean Sea (Registration code: YUC–CC–254–11) of the Unidad Multidisciplinaria de Docencia e Investigación en Sisal (UMDI-Sisal) at the Universidad Nacional Autónoma de México (UNAM), and in the American Museum of Natural History (AMNH, accession number 65822). We followed the taxonomic classification and synonymies implemented in Fautin (2013) with modifications from Rodríguez et al. (2012). Taxa are arranged in families in alphabetical order. The diagnosis of each species is based on the features observed in the collected specimens. The synonym list for each species only contains reference to the first citation of the species by a particular name. The number of specimens examined of each species per locality is indicated in the material examined. Figure 1 displays the coral reef localities sampled in this study. Table 1 indicates previous and new records of the species observed and collected at each coral reef locality; Table 2 includes size ranges of length and width of cnidae capsules for each studied species.

## Results

### Systematic treatment

#### Order Actiniaria Hertwig, 1882
Suborder Nynantheae Carlgren, 1899
Infraorder Thenaria Carlgren, 1899
Superfamily Actinoidea Rafinesque, 1815
Family Actiniidae Rafinesque, 1815
Genus *Anemonia* Risso, 1826

##### Anemonia sargassensis

Hargitt, 1908

http://species-id.net/wiki/Anemonia_sargassensis

Figure 3 , Table 2

Anemonia sargassensis Hargitt 1908: 117–118.
Anemonia antillensis Pax 1924: 94, 99–100, 119.
Anemonia sargassiensis [sic]: Carlgren 1949: 50.

###### Material examined.

Alacranes reef (22°31'35"N, 89°46'05"W; two specimens), Cayo Arenas reef (22°07'05"N, 91°24'17"W; three specimens), La Gallega reef (19°13'20"N, 96°07'39"W; two specimens), Ingenieros reef (19°08'41"N, 96°05'22"W; two specimens).

###### Diagnosis.

**Figure 3.**
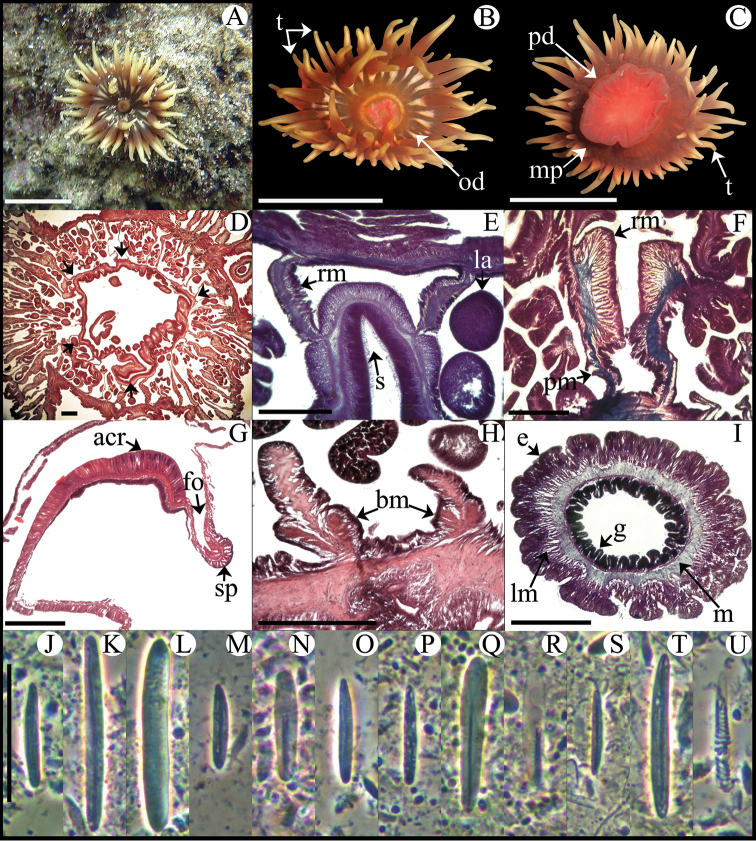
*Anemonia sargassensis*. **A** Live specimen in natural habitat **B** Oral view **C** Pedal disc view **D** Cross section through distal column showing mesenteries; arrows indicate siphonoglyphs **E** Detail of cross section through distal column showing a siphonoglyph **F** Detail of retractor and parietobasilar muscles **G** Longitudinal section through margin showing acrorhagi and marginal sphincter muscle **H** Longitudinal section through base showing basilar muscles **I** Cross section through tentacle **J–U** Cnidae.– acrorhagi: **J** small basitrich **K** basitrich **L** holotrich; actinopharynx: **M** small basitrich **N** microbasic *p*-mastigophore; column: **O** basitrich; filaments: **P** basitrich **Q** microbasic *b*-mastigophore **R** microbasic *p*-mastigophore; tentacle: **S** small basitrich **T** basitrich **U** spirocyst. Abbreviations.– acr: acrorhagi, bm: basilar muscle, e: epidermis, fo: fosse, g: gastrodermis, la: larvae, lm: longitudinal muscle, m: mesoglea, mp: marginal projection, od: oral disc, pd: pedal disc, pm: parietobasilar muscle, rm: retractor muscle, s: siphonoglyph, sp: sphincter, t: tentacle. Scale bars: **A–C**: 10 mm; D–I: 200 μm; **J–U**: 25 μm.

Fully expanded oral disc and tentacles 9–50 mm in diameter. Oral disc smooth, 4–22 mm in diameter, wider than column, dark-orange, brownish, greenish or dark-red, with white or yellowish endocoelic radial stripes tapering from tentacle bases (Figure 3A, B); mouth bright orange or pink (Figure 3B). Tentacles hexamerously arranged in 4–5 cycles (48–76 in number), moderately long (to 6–19 mm length), smooth, slender, tapering distally, inner ones longer than outer ones, contractile, dark-orange to reddish, sometimes with whitish or yellowish tips and pink or purple flashes (Figure 3A–C). Fossa well marked (Figure 3G). Poorly marked endocoelic marginal projections, 17–35, forming acrorhagi (Figure 3G), with holotrichs and basitrichs. Column cylindrical, short, smooth, 5–11 mm in diameter and 5–12 mm in height, dark-orange to dark-red. Pedal disc well-developed, 6–16 mm in diameter, wider than column (Figure 3C), bright-orange or pink. Mesenteries irregularly arranged in four cycles: first and second cycles perfect, others imperfect; more mesenteries proximally than distally (82–89 and 44–48 pairs respectively in specimens examined). Directives absent, 5–6 siphonoglyphs in specimens examined (Figure 3D, E). Gametogenic tissue not observed in specimens examined. Larvae observed in coelenteron of one specimen examined (Figure 3E). Retractor muscles diffuse to restricted; parietobasilar muscles weak with short mesogleal pennon (Figure 3F). Basilar muscles well-developed (Figure 3H). Marginal sphincter muscle endodermal, diffuse (Figure 3G). Longitudinal muscles of tentacles ectodermal (Figure 3I). Zooxanthellae present. Cnidom: basitrichs, holotrichs, microbasic *b*- and *p*-mastigophores and spirocysts (Figure 3J–U; see Table 2).

**Table 2.** Size and distribution of preserved cnidae from specimens examined. “m_l_” and “m_w_” are the means (length and width respectively), “d_l_” and “d_w_” are the standard deviations (length and width respectively), all in µm. “#^1^” and “#^2^” is the number of capsules measured per each specimen examined, “p” is the proportion of animals examined with respective to the type of cnida present.

**Species**	**Tissue**	**Cnida**	**Capsule length (µm)**	**m_l_**	**d_l_**	**Capsule width (µm)**	**m_w_**	**d_w_**	**#^1^**	**#^2^**	**p**
*Anemonia sargassensis*	Tentacle	Basitrich	8.7–20.2	16.1	2.2	1.6–3.3	2.1	0.2	24	21	2/2
		Basitrich	21.0–36.8	30.8	3.6	2.4–3.6	3.0	0.2	23	21	2/2
		Spirocyst	15.1–40.0	25.3	7.0	2.2–3.5	2.9	0.3	21	20	2/2
	Actinopharynx	Basitrich	13.9–33.5	24.9	3.9	2.1–4.0	3.2	0.4	20	26	2/2
		Microbasic *p*-mastigophore	16.8–24.9	19.7	2.2	3.3–5.6	4.5	0.6	6	9	2/2
	Column	Basitrich	13.3–22.6	18.3	2.4	2.1–3.0	2.5	0.2	21	20	2/2
	Acrorhagi	Basitrich	14.6–26.4	20.1	3.0	2.1–3.2	2.4	0.1	20	22	2/2
		Basitrich	27.8–43.7	35.8	3.6	2.5–3.5	3.1	0.1	21	21	2/2
		Holotrich	31.1–42.4	36.8	2.6	4.4–6.9	5.1	0.4	22	20	2/2
	Filament	Basitrich	12.9–32.7	19.2	5.2	1.8–3.0	2.4	0.2	20	27	2/2
		Microbasic *b*-mastigophore	24.6–33.9	28.6	2.5	3.7–6.0	4.7	0.6	22	21	2/2
		Microbasic *p*-mastigophore	15.0–24.5	20.4	2.3	3.7–5.9	4.8	0.5	21	22	2/2
*Anthopleura pallida*	Tentacle	Basitrich	12.6–20.6	16.8	2.0	1.7–2.6	2.1	0.2	23	21	2/2
		Spirocyst	11.8–19.2	16.0	1.4	2.3–3.6	2.9	0.3	28	21	2/2
	Actinopharynx	Basitrich	15.0–27.0	21.6	3.1	1.8–3.1	2.5	0.3	23	24	2/2
		Basitrich	10.1–18.0	14.0	1.7	1.5–2.4	1.9	0.1	15	22	2/2
		Spirocyst	11.2–19.5	16.2	1.9	2.3–3.7	2.8	0.3	12	21	2/2
		Microbasic *b*-mastigophore	20.9–28.3	24.6	2.7	2.8–4.4	3.3	0.5	5	1	2/2
		Microbasic *p*-mastigophore	13.4–23.5	19.4	3.9	3.8–5.0	4.3	0.5	1	4	2/2
	Column	Basitrich	14.7–19.3	17.2	1.2	2.8–4.2	3.2	0.3	22	22	2/2
		Basitrich	8.7–17.1	14.2	1.6	1.4–2.4	2.0	0.1	26	21	2/2
		Spirocyst	10.3–14.7	12.6	1.5	2.3–2.6	2.5	0.1	5	1	2/2
	Acrorhagi	Basitrich	12.2–25.3	16.6	2.7	1.7–2.6	2.1	0.2	25	23	2/2
		Basitrich	7.6–14.9	11.8	1.4	1.4–2.1	1.7	0.1	23	0	1/2
		Spirocyst	11.3–23.9	17.8	2.6	1.9–3.5	2.6	0.4	22	20	2/2
		Holotrich	17.9–39.3	31.8	3.8	2.4–4.7	3.6	0.5	29	25	2/2
		Holotrich	21.1–36.5	27.9	4.2	2.3–3.3	2.8	0.2	24	0	1/2
		Microbasic *p*-mastigophore	16.5–17.5	17.0	0.7	2.8–4.1	3.4	0.8	2	0	1/2
	Filament	Basitrich	13.1–33.7	17.3	3.9	1.9–3.0	2.3	0.3	15	7	2/2
		Basitrich	9.2–18.5	14.1	2.1	1.2–2.3	2.0	0.2	3	20	2/2
		Spirocyst	10.9–19.2	15.5	2.1	1.9–3.5	2.6	0.3	20	4	2/2
		Microbasic *b*-mastigophore	15.5–28.0	24.9	2.4	3.1–4.6	3.7	0.3	7	20	2/2
		Holotrich	29.7–33.5	31.6	2.6	2.8–3.0	2.9	0.1	2	0	1/2
		Microbasic *p*-mastigophore	17.2–23.4	20.5	2.2	4.2–4.8	4.5	0.2	1	4	2/2
*Bunodosoma cavernatum*	Tentacle	Basitrich	10.7–29.5	21.0	4.7	1.6–3.4	2.1	0.4	21	22	2/2
		Spirocyst	13.2–22.6	16.8	2.3	1.7–3.7	2.5	0.6	20	23	2/2
	Actinopharynx	Basitrich	21.0–27.2	24.7	1.2	2.8–3.5	3.2	0.1	22	20	2/2
		Microbasic *p*-mastigophore	16.3–21.1	18.5	1.5	3.7–6.0	4.9	0.5	4	22	2/2
	Column	Basitrich	14.7–19.8	16.8	1.2	1.8–2.5	2.2	0.1	20	20	2/2
		Basitrich	20.8–28.4	24.8	1.6	2.5–3.9	3.0	0.2	31	21	2/2
	Acrorhagi	Basitrich	17.2–28.8	22.8	3.5	2.1–3.5	2.7	0.4	21	20	2/2
		Holotrich	26.6–45.1	35.0	3.7	3.1–5.8	4.0	0.5	22	20	2/2
	Filament	Basitrich	11.9–28.5	23.9	4.7	1.6–4.0	3.0	0.5	6	21	2/2
		Microbasic *b*-mastigophore	20.5–37.4	28.0	4.3	4.2–8.9	6.2	1.7	30	22	2/2
		Microbasic *p*-mastigophore	14.4–23.1	18.7	2.9	3.2–6.7	4.6	0.9	20	21	2/2
*Isoaulactinia stelloides*	Tentacle	Basitrich	14.1–23.6	18.5	2.8	1.9–2.8	2.4	0.2	21	21	2/2
		Macrobasic *p*-mastigophore	16.0–25.6	22.0	1.6	5.1–9.2	7.3	0.9	23	22	2/2
		Spirocyst	12.2–22.2	17.4	2.6	1.9–3.0	2.4	0.2	21	21	2/2
	Actinopharynx	Basitrich	11.7–18.5	13.7	1.7	1.6–2.7	2.1	0.2	21	21	2/2
		Basitrich	16.6–34.1	26.4	2.9	2.3–3.2	2.9	0.2	29	22	2/2
		Macrobasic *p*-mastigophore	21.1–26.9	24.4	1.4	6.3–8.3	7.6	0.5	4	20	2/2
		Microbasic *p*-mastigophore	18.0–28.6	25.2	2.7	4.1–5.7	4.9	0.4	10	5	2/2
		Microbasic *b*-mastigophore	15.2–33.2	26.9	5.7	2.8–4.0	3.4	0.3	1	6	2/2
		Long, curved basitrich	18.9–32.8	24.9	4.8	1.6–2.2	1.9	0.2	6	6	2/2
	Column	Basitrich	11.8–15.7	13.5	0.9	1.9–2.8	2.3	0.1	23	21	2/2
		Macrobasic *p*-mastigophore	22.2–27.7	24.5	1.2	5.2–7.5	6.3	0.5	26	20	2/2
		Long, curved basitrich	25.1–31.3	28.2	4.3	2.2–2.3	2.3	0.1	2	0	1/2
	Marginal projection	Basitrich	11.1–13.8	12.3	0.7	1.8–2.8	2.3	0.2	26	20	2/2
		Macrobasic *p*-mastigophore	20.1–25.9	22.7	1.4	5.2–8.5	6.5	0.8	32	20	2/2
	Filament	Basitrich	10.8–15.5	13.4	1.1	1.6–2.2	1.9	0.1	24	20	2/2
		Basitrich	17.6–31.8	22.2	3.4	1.7–3.1	2.3	0.3	20	21	2/2
		Macrobasic *p*-mastigophore	23.3–29.3	26.0	1.4	5.9–8.1	7.1	0.4	20	20	2/2
		Microbasic *p*-mastigophore	17.6–32.3	25.9	3.3	3.9–6.0	4.7	0.4	15	23	2/2
		Microbasic *b*-mastigophore	29.3–39.7	34.1	2.4	3.2–4.9	3.9	0.4	20	22	2/2
		Long, curved basitrich	17.0–29.7	24.1	5.5	1.5–2.2	1.9	0.3	4	0	1/2
*Lebrunia coralligens*	Tentacle	Basitrich	12.3–33.5	26.2	4.8	1.7–2.6	2.2	0.2	20	24	2/2
		Spirocyst	17.1–29.9	23.8	3.5	2.8–5.5	4.1	0.6	2	21	2/2
		Microbasic *p*-amastigophore	11.8–14.6	13.2	1.1	2.5–3.1	2.7	0.2	4	0	1/2
		Microbasic *p*-amastigophore	29.0–68.7	48.6	9.0	4.4–7.1	5.6	0.6	20	21	2/2
	Pseudotentacle	Basitrich	8.9–26.8	15.4	4.3	1.7–2.8	2.2	0.2	22	23	2/2
		Microbasic *p*-amastigophore	37.2–67.8	51.5	5.7	10.8–15.7	13.0	1.8	25	21	2/2
		Microbasic *p*-amastigophore	11.7–25.9	17.2	2.8	2.3–4.6	3.3	0.4	20	20	2/2
	Actinopharynx	Microbasic *p*-amastigophore	10.7–21.6	13.7	2.0	2.3–3.7	2.7	0.3	20	23	2/2
		Microbasic *p*-amastigophore	18.8–45.1	34.6	7.9	3.4–6.3	5.1	0.6	21	20	2/2
	Column	Basitrich	9.0–14.0	10.9	1.0	1.6–2.6	2.1	0.2	24	20	2/2
		Microbasic *p*-amastigophore	12.1–23.5	14.8	1.8	2.7–4.0	3.3	0.3	23	21	2/2
	Filament	Microbasic *p*-amastigophore	11.2–17.3	13.6	1.2	2.2–3.3	2.7	0.3	20	20	2/2
		Microbasic *p*-amastigophore	29.1–46.5	37.1	4.0	4.4–6.2	5.4	0.4	20	10	2/2
*Actinoporus elegans*	Tentacle	Basitrich	15.8–20.8	17.4	2.2	2.4–3.0	2.7	0.2	4	0	1/1
		Spirocyst	26.6–37.6	32.8	3.2	2.2–2.9	2.6	0.1	23	0	1/1
	Actinopharynx	Basitrich	25.6–32.5	27.9	1.7	3.5–4.8	4.2	0.3	21	0	1/1
		Microbasic *p*-mastigophore	29.8–34.9	31.9	1.4	6.6–9.0	7.8	0.5	22	0	1/1
	Column	Basitrich	10.1–24.9	17.8	3.7	1.5–2.6	2.1	0.2	22	0	1/1
	Filament	Basitrich	16.1–24.5	21.3	2.2	2.1–3.1	2.6	0.2	20	0	1/1
		Microbasic *p*-mastigophore	25.7–30.2	27.8	1.1	5.3–6.9	6.0	0.4	20	0	1/1
*Calliactis tricolor*	Tentacle	Basitrich	12.9–16.3	15.0	0.8	1.4–2.5	1.7	0.2	21	6	2/2
		Spirocyst	16.9–29.1	22.9	3.1	3.0–4.9	3.9	0.6	0	21	1/2
	Actinopharynx	Basitrich	13.3–25.5	19.3	4.4	1.4–3.3	2.3	0.4	24	21	2/2
		Microbasic *p*-mastigophore	13.4–18.2	16.0	1.0	2.3–3.1	2.6	0.2	20	0	1/2
	Column	Basitrich	8.0–16.5	11.4	2.4	1.3–2.4	1.8	0.3	20	20	2/2
	Filament	Basitrich	13.7–26.2	19.5	4.1	1.9–3.0	2.3	0.2	20	21	2/2
		Basitrich	9.2–12.7	10.7	0.8	1.4–1.8	1.7	0.1	0	22	1/2
		Microbasic *p*-mastigophore	14.2–24.1	17.6	2.8	2.3–4.6	3.3	0.7	22	21	2/2
	Acontia	Basitrich	13.6–25.3	19.5	3.9	2.0–3.4	2.7	0.4	20	21	2/2

###### Natural history.

*Anemonia sargassensis* inhabits shallow waters of the lagoon reef zone, often above *Thalassia testudinum* blades, but is also found under stones and coral gravel, between 0.5–2 m. It is often reported on floating *Sargassum* (Carlgren and Hedgpeth 1952). Asexual propagation by longitudinal fission is common (Carlgren and Hedgpeth 1952) and bifurcated tentacles can occur (Hargitt 1908, 1912, Pax 1924, Corrêa 1964, present study).

###### Distribution.

Western Atlantic, from the northern coast of USA and Caribbean Sea, to the northern coast of Brazil (Carlgren and Hedgpeth 1952, Varela 2002, Zamponi et al. 1998).

###### Remarks.

Of the 20 valid species of *Anemonia*, four species have been recorded in the Gulf of Mexico and Caribbean Sea (Fautin 2013): *Anemonia sargassensis*, *Anemonia melanaster* (Verrill, 1901), *Anemonia depressa* Duchassaing & Michelotti, 1860, and *Anemonia elegans* Verrill, 1901. The anatomy described for *Anemonia sargassensis* is conflicting mainly in the presence of directives, siphonoglyphs, and marginal projections (e.g. Hargitt 1908, 1912, Pax 1924, Field 1949, Carlgren and Hedgpeth 1952). Just as in Field (1949), Carlgren and Hedgpeth (1952), and Corrêa (1964), we did not find directives in our specimens but 5–6 siphonoglyphs were present. Although some authors suggest that *Anemonia sargassensis* and *Anemonia melanaster* are synonymous (Cairns et al. 1986, Ocaña and den Hartog 2002, Wirtz et al. 2003), further studies are necessary to establish the current taxonomic status of both species. Differences between the other two species of the genus in the region, *Anemonia depressa* and *Anemonia elegans*, and *Anemonia sargassensis* are not clear based on the scarce information available and also require further revision.

#### Genus *Anthopleura* Duchassaing & Michelotti, 1860

##### Anthopleura pallida

Duchassaing & Michelotti, 1864

http://species-id.net/wiki/Anthopleura_pallida

Figure 4 , Table 2

Anthopleura pallida Duchassaing and Michelotti 1864: 32–33; Pl. V, fig. 10.
Anthopleura Pallida [sic]: Duchassaing 1870: 20.
Gyractis pallida non Boveri 1893: 251–252.
Actinioides pallida : Duerden 1897: 453.
Actinoides pallida : Verrill 1900: 558.
Bunodactis stelloides catenulata Verrill 1905: 263.
Anthopleura pallida non Carlgren, 1949: 53.
Anthopleura catenulata : Cairns, den Hartog and Arneson 1986: 177–178; Pl. 51.

###### Material examined.

Alacranes reef (22°22'54"N, 89°40'59"W; four specimens).

###### Diagnosis.

**Figure 4.**
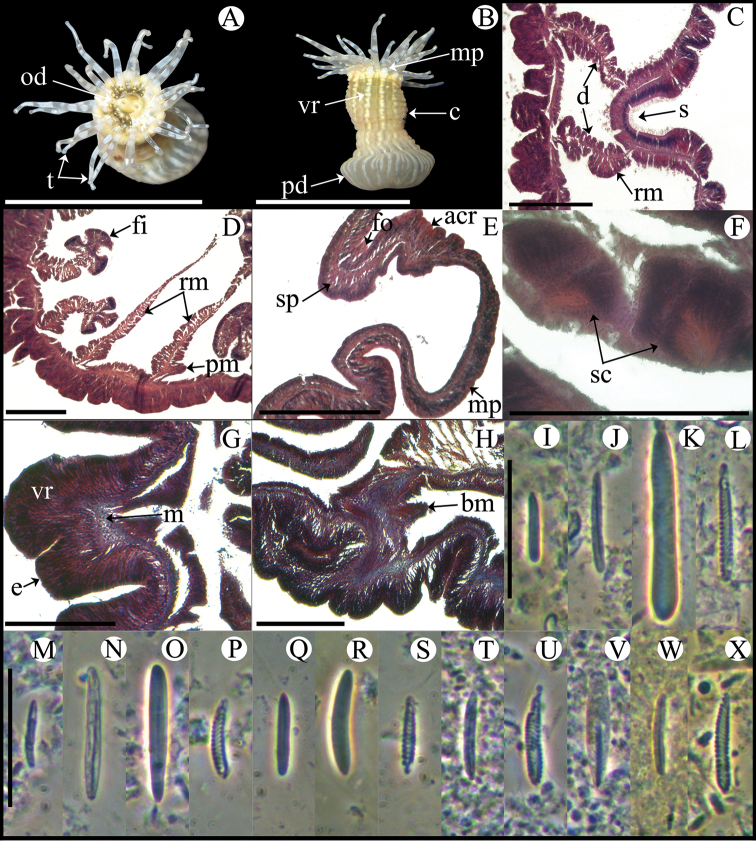
*Anthopleura pallida*. **A** Oral view **B** Lateral view **C** Detail of directives and siphonoglyph **D** Cross section through proximal column **E** Longitudinal section through margin showing acrorhagi and marginal sphincter muscle **F** Detail of spermatic cysts **G** Longitudinal section through distal column showing one verruca **H** Longitudinal section through base showing basilar muscles **I–X** Cnidae.– acrorhagi: **I** small basitrich **J** basitrich **K** holotrich **L** spirocyst; actinopharynx: **M** small basitrich **N** basitrich **O** microbasic *b*-mastigophore **P** spirocyst; column: **Q** small basitrich **R** basitrich **S** spirocyst; filament: **T** basitrich **U** spirocyst **V** microbasic *p*-mastigophore; tentacle: **W** basitrich **X** spirocyst. Abbreviations.– acr: acrorhagi, bm: basilar muscle, c: column, d: directives, fo: fosse, mp: marginal projection, od: oral disc, pd: pedal disc, pm: parietobasilar muscle, rm: retractor muscle, s: siphonoglyph, sc: spermatic cyst, sp: sphincter, t: tentacles, vr: verruca. Scale bars: **A–B**: 10 mm; **C–H**: 200 μm; **I–X**: 25 μm.

Fully expanded oral disc and tentacles 10–19 mm in diameter. Oral disc narrow, smooth, 3–8 mm in diameter, pale green or gray (Figure 4A). Tentacles hexamerously arranged in three cycles (24 in number), smooth, slender, relatively short (to 4–9 mm), tapering distally, inner ones longer than outer ones, contractile, whitish or gray, translucent, oral side with opaque white roundish spots (Figure 4A, B). Fosse well marked (Figure 4E). Column cylindrical, relatively elongate, 3–6 mm in diameter and 6–12 mm in height, with 12 longitudinal rows of verrucae from mid-column to distal margin (Figure 4B, G). Twelve endocoelic marginal projections forming acrorhagi (Figure 4B, E) with holotrichs, basitrichs, microbasic *p*-mastigophores, and spirocysts. Pedal disc well-developed, 4–8 mm in diameter, slightly wider than column (Figure 4B). Pedal disc and column white to pale green (Figure 4B). Mesenteries hexamerously arranged in 2–3 cycles: only first cycle perfect or first two cycles perfect and third imperfect; same number of mesenteries distally and proximally (12–32 pairs in specimens examined). Only first two cycles fertile (except directives); gonochoric (?), only spermatic cysts observed in specimens examined (Figure 4F). Two pairs of directives each attached to a well-developed siphonoglyph (Figure 4C). Retractor muscles diffuse; parietobasilar muscles well-developed with short mesogleal pennon (Figure 4D). Basilar muscles well-developed (Figure 4H). Marginal sphincter muscle endodermal, weak and diffuse (Figure 4E). Longitudinal muscles of tentacles ectodermal. Cnidom: basitrichs, microbasic *b*- and *p*-mastigophores, holotrichs, and spirocysts (Figure 4I–X; see Table 2).

###### Natural history.

*Anthopleura pallida* inhabits the intertidal to shallow subtidal zone attached to coral on sandy shores, at 0.5 m. It is azooxanthellate and it broadcast spawns (Daly and den Hartog 2004).

###### Distribution.

Western Atlantic, from Bermuda (Verrill 1900) to Virgin Islands (Duchassaing and Michelotti 1864). This is the first record for the coast of Mexico; found in Alacranes reef (see Table 1).

###### Remarks.

Currently there are three valid species of *Anthopleura* in the Gulf of Mexico and Caribbean Sea: *Anthopleura krebsi* (Duchassaing & Michelotti, 1860), *Anthopleura texaensis* (Carlgren and Hedgpeth, 1952), and *Anthopleura pallida* (Daly and den Hartog 2004). *Anthopleura pallida* is distinguished mainly in column color and shape, and the arrangement of verrucae in rows, only present from the margin to the mid-column (Daly and den Hartog 2004). However, in *Anthopleura krebsi* and *Anthopleura texaensis*, the column is stout rather than elongate, and the verrucae are arranged in rows along the entire column length, from margin to limbus (Daly and den Hartog 2004). Although we found the marginal sphincter muscle diffuse rather than circumscribed-diffuse, all other features including external and internal anatomy and cnidae fit well with the redescription of *Anthopleura pallida* by Daly and den Hartog (2004).

#### Genus *Bunodosoma* Verrill, 1899

##### Bunodosoma cavernatum

(Bosc, 1802)

http://species-id.net/wiki/Bunodosoma_cavernatum

Figure 5 , Table 2

Actinia cavernata Bosc 1802: 221–222.
Urticina cavernata : Duchassaing 1850: 9.
Bunodes cavernata : Verrill 1864: 17–18.
Phymactis cavernata : Andres 1883: 448.
Bunodosoma cavernata : Verrill 1899: 45.
Anthopleura cavernata : Cary 1906: 51.
Bunodosma cavernata : Daly 2003: 92.

###### Material examined.

La Gallega reef (19°13'20"N, 96°07'39"W; thirteen specimens), Ingenieros reef (19°08'41"N, 96°05'22"W; two specimens).

###### Diagnosis.

**Figure 5.**
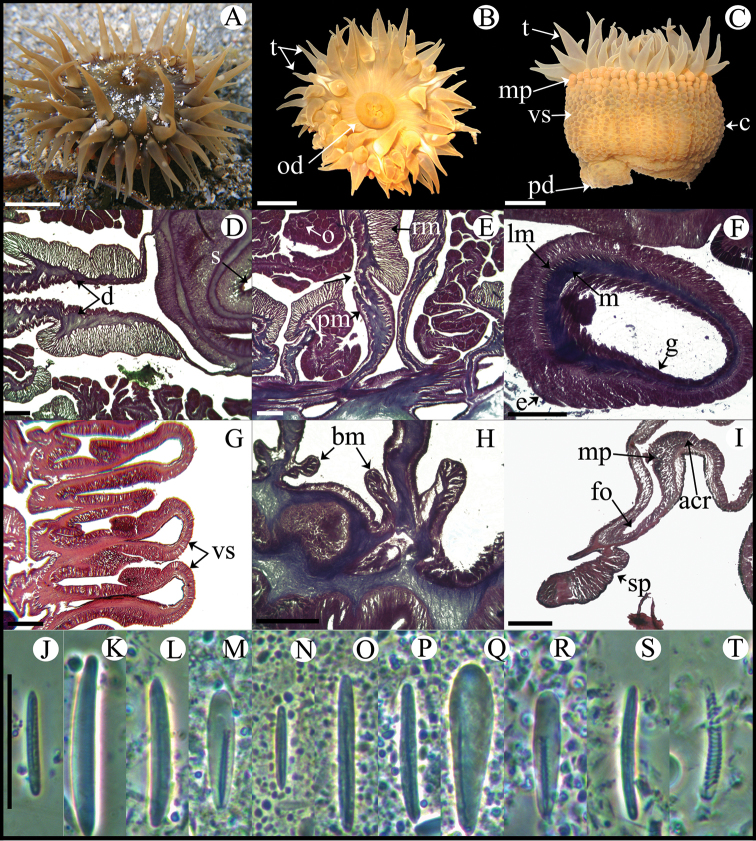
*Bunodosoma cavernatum*. **A** Live specimen in natural habitat **B** Oral view **C** Lateral view **D** Detail of directives; notice siphonoglyph **E** Cross section through proximal column showing oocytes **F** Cross section through tentacle **G** Longitudinal section through column showing vesicles **H** Longitudinal section though base showing basilar muscles **I** Longitudinal section through margin showing acrorhagi and marginal sphincter muscle **J–T** Cnidae.– acrorhagi: **J** basitrich **K** holotrich; actinopharynx: **L** basitrich **M** microbasic *p*-mastigophore; column: **N** small basitrich **O** basitrich; filament: **P** basitrich **Q** microbasic *b*-mastigophore **R** microbasic *p*-mastigophore; tentacle **S** basitrich **T** spirocyst. Abbreviations.– acr: acrorhagi, bm: basilar muscle, c: column, d: directives, e: epidermis, fo: fosse, g: gastrodermis, lm: longitudinal muscles, m: mesoglea, mp: marginal projection, o: oocyst, od: oral disc, pd: pedal disc, pm: parietobasilar muscle, rm: retractor muscle, s: siphonoglyph, sp: sphincter, t: tentacle, vs: vesicles. Scale bars: **A–C**: 10 mm; **D–I**: 200 μm; **J–T**: 25 μm.

Fully expanded oral disc and tentacles to 20–38 mm in diameter. Oral disc 10–22 mm in diameter, smooth, brown-yellowish, brown-reddish or pale olive-green, sometimes with white or yellowish radial stripes in endocoelic spaces of first two or three tentacular cycles (Figure 5A, B). Tentacles hexamerously arranged in five cycles (about 96 in number), smooth, simple, conical, moderately long (3–5 mm in length), tapering distally, inner ones longer than outer ones, contractile, olive-green, reddish or pale-orange (Figure 5A, B), often with white or yellowish spots on oral side and sometimes with purple flashes. Deep fosse (Figure 5I). Forty-eight endocoelic rounded marginal projections forming acrorhagi (Figure 5C, I) with holotrichs and basitrichs. Column cylindrical, 12–22 in diameter and 7–15 mm in height, densely covered with rounded vesicles, arranged in 96 longitudinal rows from margin to limbus (Figure 5C, G). Pedal disc well-developed, 12–19 mm in diameter (Figure 5C). Column and pedal disc light-brown, orange, reddish, yellowish or olive-green. Mesenteries hexamerously arranged in four cycles (48 pairs in specimens examined): first, second and some mesenteries of third cycle perfect, others imperfect; same number of mesenteries distally and proximally. All mesenteries fertile (except directives); gonochoric; oocytes and spermatic cysts well-developed in specimens collected in January and May (Figure 5E). Two pairs of directives each attached to a well-developed siphonoglyph (Figure 5D). Retractor muscles strong and restricted; parietobasilar muscles well-developed with a relatively long free mesogleal pennon (Figure 5E). Basilar muscles well-developed (Figure 5H). Marginal sphincter muscle endodermal, strong and circumscribed (Figure 5I). Longitudinal muscles of tentacles ectodermal (Figure 5F). Zooxanthellae present. Cnidom: basitrichs, microbasic *b*- and *p*-mastigophores, holotrichs and spirocysts (Figure 5J–T; see Table 2).

###### Natural history.

*Bunodosoma cavernatum* inhabits shallow waters, attached to rocks and coral rubble, in the lagoon zone; between 2–6 m.

###### Distribution.

Western Atlantic, from North Caroline to Barbados; along the Caribbean Sea and Gulf of Mexico (Carlgren and Hedgpeth 1952); and Caroline Islands, Micronesia (Bosc 1802). This is the first record for the coast of Mexico; found in the VRS (see Table 1).

###### Remarks.

Currently four valid species of *Bunodosoma* have been reported in the Gulf of Mexico and Caribbean Sea (González-Muñoz et al. 2012, Fautin 2013): *Bunodosoma cavernatum*, *Bunodosoma granuliferum* (Le Sueur, 1817), *Bunodosoma kuekenthali* Pax, 1910, and *Bunodosoma sphaerulatum* Duerden, 1902. *Bunodosoma cavernatum* differs from *Bunodosoma granuliferum* because it lacks the distinct chromatic pattern of the column with alternating pale and dark longitudinal bands but also based on molecular evidence (reviewed in González-Muñoz et al. 2012). Our specimens show that the circumscribed marginal sphincter muscle tends to split in two parts as suggested by Carlgren (1952) (Figure 5I). The distinction between *Bunodosoma sphaerulatum* and *Bunodosoma kuekenthali* and their Caribbean congeners are not clear based on the information available.

#### Genus *Isoaulactinia* Belém, Herrera-Moreno & Schlenz, 1996

##### Isoaulactinia stelloides

(McMurrich, 1889)

http://species-id.net/wiki/Isoaulactinia_stelloides

Figure 6 , Table 2

Aulactinia stelloides McMurrich 1889: 28–31.
Aulactinia stella : Duerden 1897: 454–455.
Bunodella stelloides : Verrill 1899: 43–44.
Bunodes stella : Duerden 1898: 455.
Bunodactis stelloides : Verrill 1900: 556.
Anthopleura catenulata : Cairns, den Hartog and Arneson 1986: 177–178; Pl. 51.
Anthopleura carneola : Cairns, den Hartog and Arneson 1986: 177–178; Pl. 51.
Isoaulactinia stelloides : Belém, Herrera-Moreno and Schlenz 1996: 77–88.

###### Material examined.

La Gallega reef (19°13'20"N, 96°07'39"W; six specimens).

###### Diagnosis.

**Figure 6.**
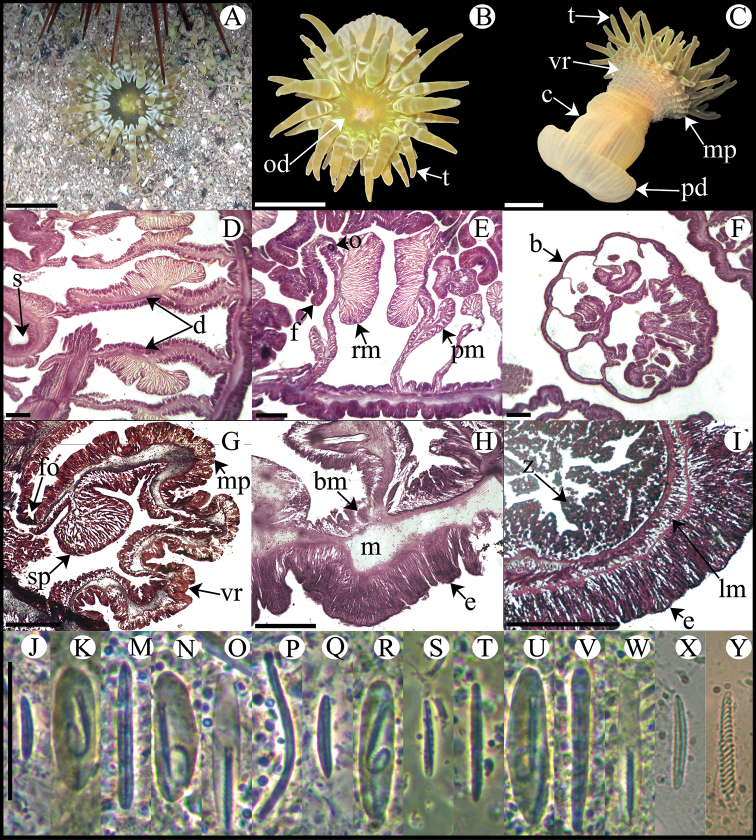
*Isoaulactinia stelloides*. **A** Live specimen in natural habitat **B** Oral view **C** Lateral view **D** Detail of directives showing a siphonoglyph **E** Cross section through proximal column **F** Detail of brooded juvenile **G** Longitudinal section through margin showing marginal sphincter muscle and marginal projection **H** Longitudinal section though base showing basilar muscles **I** Cross section through tentacle **J–Y** Cnidae.– marginal projection: **J** small basitrich **K** macrobasic p-mastigophore; actinopharynx: **M** basitrich **N** macrobasic p-mastigophore **O** microbasic p-mastigophore **P** long, curved basitrich; column: **Q** small basitrich **R** macrobasic p-mastigophore; filament: **S** small basitrich **T** basitrich **U** macrobasic p-mastigophore **V** microbasic b-mastigophore **W** microbasic p-mastigophore; tentacle: **X** basitrich **Y** spirocyst. Abbreviations.– b: brooded juvenile, bm: basilar muscle, c: column, d: directives, e: epidermis, fo: fosse, lm: longitudinal muscle, m: mesoglea, mp: marginal projection, o: oocyst, od: oral disc, pd: pedal disc, pm: parietobasilar muscle, rm: retractor muscle, s: siphonoglyph, sp: sphincter, t: tentacle, vr: verrucae, z: zooxanthellae. Scale bars: **A–C**: 10 mm; **D–I**: 200 μm; **J–Y**: 25 μm.

Fully expanded oral disc and tentacles to 24–38 mm in diameter. Oral disc smooth, slightly wider than column, 9–11 mm in diameter, light- or olive-green, sometimes with small white stripes near tentacles bases (Figure 6A, B). Tentacles hexamerously arranged in four cycles (about 48 in number), simple, smooth, moderately long (9–14 mm in length), conical, tapering distally, inner ones longer than outer ones, contractile, olive-green with white bands along entire length (Figure 6A, B). Deep fosse (Figure 6G). Twenty-four endocoelic marginal projections (Figure 6C, G) with basitrichs and macrobasic *p*-mastigophores. Column cylindrical, 8–12 in diameter and 13–22 mm in height, with approximately 48 longitudinal rows of verrucae along entire column, but more conspicuous distally (Figure 6C). Pedal disc well-developed, 9–16 mm in diameter (Figure 6C). Column, verrucae, and pedal disc light-brown or beige (Figure 6C). Mesenteries hexamerously arranged in three cycles (24 pairs in specimens examined): all cycles perfect; same number of mesenteries distally and proximally. First and second cycles fertile (except directives); hermaphroditic (?), only oocytes observed in specimens examined (Figure 6E). Developing polyps in coelenteron (Figure 6F). Two pairs of directives each attached to a well-developed siphonoglyph (Figure 6D). Retractor muscles strong and restricted; parietobasilar muscles well-developed with relatively long and thick free mesogleal pennon (Figure 6E). Basilar muscles well-developed (Figure 6H). Marginal sphincter muscle endodermal, strong and circumscribed, palmate (Figure 6G). Longitudinal muscles of tentacles ectodermal (Figure 6I). Zooxanthellae present. Cnidom: basitrichs, microbasic *b*-mastigophores, macrobasic and microbasic *p*-mastigophores, and spirocysts (Figure 6J–Y; see Table 2).

###### Natural history.

*Isoaulactinia stelloides* inhabits shallow waters in the lagoon reef zone, at 1–2 m, near *Actinostella flosculifera*, *Stichodactyla helianthus*, and the zoanthid *Palythoa caribaeorum* (Duchassaing & Michelotti, 1860). It lives with the column burrowed in the sand but the pedal disc attached to rocks and coral rubble. Although we only observed developing oocytes in the two specimens histologically examined, *Isoaulactinia stelloides* has been reported as a simultaneous hermaphroditic, internally brooding, often with developing polyps in the coelenteron (Belém et al. 1996, Daly and den Hartog 2004); the latter have also been observed in the present study (Figure 6F).

###### Distribution.

Western Atlantic, from Bermuda to Barbados, and along the Caribbean Sea (Belém et al. 1996, Daly and den Hartog 2004). This is the first record for the coast of Mexico; found in the VRS (see Table 1).

###### Remarks.

Currently *Isoaulactinia* has two valid species (Daly 2004, Fautin 2013): *Isoaulactinia hespervolita* Daly, 2004, and *Isoaulactinia stelloides*. According to Daly (2004) *Isoaulactinia hespervolita* differs from *Isoaulactinia stelloides* in having an unmarked oral disc and tentacles, spinose holotrichs in the column and being gonochoric rather than hermaphroditic. In addition, *Isoaulactinia hespervolita* has a reddish-orange to greenish-brown column, oral disc and tentacles; approximately 80 tentacles arranged in up to five cycles, and macrobasic *p*-mastigophores only in the column and tentacles (Daly 2004). We found additional microbasic *b*-mastigophores in the actinopharynx of *Isoaulactinia stelloides* but they were not abundant (Table 2). This category of nematocyst has not been previously reported in the actinopharynx of either of the species (Belém et al. 1996, Daly and den Hartog 2004).

#### Family Aliciidae Duerden, 1895
Genus *Lebrunia* Duchassaing & Michelotti, 1860

##### Lebrunia coralligens

(Wilson, 1890)

http://species-id.net/wiki/Lebrunia_coralligens

Figure 7 ; Table 2

Hoplophoria coralligens Wilson 1890: 379–386.
Lebrunea coralligens : Duerden 1898: 456–457.
Lebrunia coralligens : Stephenson 1922: 288.

###### Material examined.

Isla Verde reef (19°13'26"N, 96°05'56"W; three specimens); Isla Sacrificios reef (19°10'36"N, 96°05'39"W; three specimens).

###### Diagnosis.

**Figure 7.**
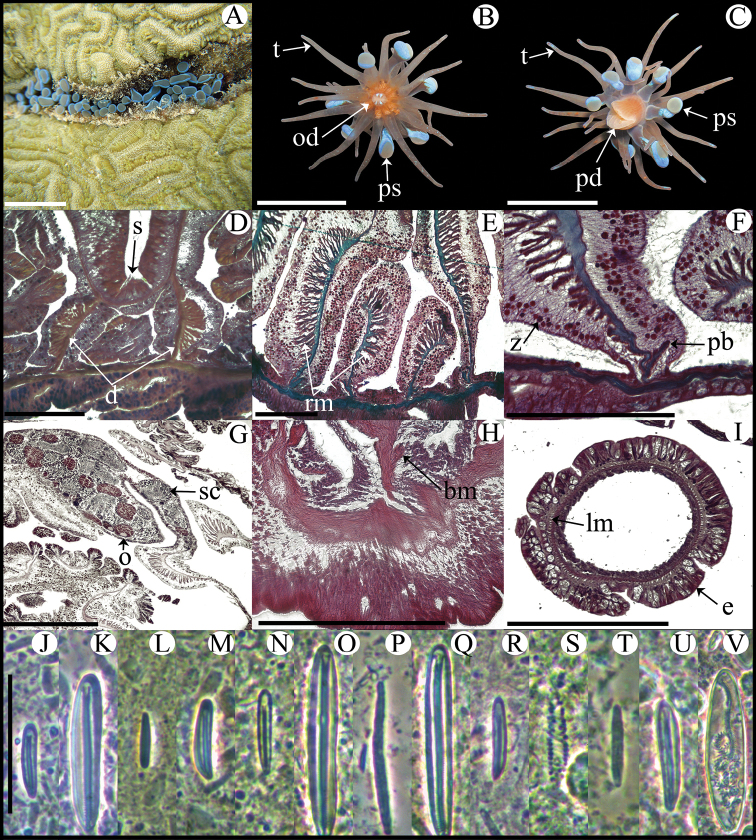
*Lebrunia coralligens*. **A** Live specimen in natural habitat **B** Oral view **C** Pedal disc view **D** Cross section through distal column showing a siphonoglyph **E** Detail of retractor muscles **F** Detail of parietobasilar muscles **G** Detail of a mesentery showing oocytes and spermatic cysts **H** Longitudinal section through base showing basilar muscles **I** Cross section through tentacle **J–V** Cnidae.– actinopharynx: **J** small microbasic *p*-amastigopore **K** microbasic *p*-amastigophore; column: **L** small basitrich **M** small microbasic *p*-amastigophore; filament: **N** small microbasic *p*-amastigophore **O** microbasic *p*-amastigophore; tentacle: **P** basitrich **Q** microbasic *p*-amastigophore **R** small microbasic *p*-amastigophore **S** spirocyst; pseudotentacle: **T** basitrich **U** microbasic *p*-amastigophore **V** macrobasic *p*-amastigophore. Abbreviations.– bm: basilar muscle, d: directives, e: epidermis, lm: longitudinal muscle, o: oocyst, od: oral disc, pd: pedal disc, pm: parietobasilar muscle, ps: pseudotentacle, rm: retractor muscle, s: siphonoglyph, sc: spermatic cyst; t: tentacle, z: zooxanthellae. Scale bars: **A–C**: 10 mm; D–H: 200 μm; I: 100 μm; **J–U**: 25 μm; **V**: 20 μm.

Fully expanded oral disc and tentacles to 18–22 mm in diameter. Oral disc smooth, 3–5 mm in diameter, beige and translucent (Figure 7B). Tentacles hexamerously arranged in 3–4 cycles (about 24–52 in number), moderately long (about 5–8 mm length), tapering distally, inner ones longer than outer ones, contractile, gray or beige, translucent, with tips whitish or yellowish and scattered bluish dots along the entire length (Figure 7B, C). Column short, smooth, 3–6 mm in diameter and 6–10 mm in height, bright-brown with faint stripes corresponding to mesenterial insertions. Column distally with 4–6 outgrowths (pseudotentacles). Pseudotentacles branched, ending in globular-shaped vesicles with batteries of macro- and micro-basic *p*-amastigophores and basitrichs; bluish with gray or brown circle in center (Figure 7A–C). Pedal disc well-developed, circular, 3–7 mm in diameter, light brown or beige, translucent (Figure 7C). Mesenteries hexamerously arranged in 2–3 cycles (12–24 pairs in specimens examined): first cycle perfect and sterile, others imperfect and fertile; more mesenteries proximally than distally (two and three cycles, respectively). Hermaphroditic (Figure 7G). Two pairs of directives each attached to a well-developed siphonoglyph (Figure 7D). Retractor muscles diffuse, strong; parietobasilar muscles with short and thick mesogleal pennon (Figure 7E, F). Basilar muscles relatively poorly developed (Figure 7H). Marginal sphincter muscle absent. Ectodermal longitudinal muscles in distal column. Longitudinal muscles of tentacles ectodermal (Figure 7I). Zooxanthellae present (Figure 7F). Cnidom: basitrichs, macrobasic and microbasic *p*-amastigophores, and spirocysts (Figure 7J–V; see Table 2).

###### Natural history.

*Lebrunia coralligens* inhabits narrow fissures of live coral with only the end of the pseudotentacles visible, between 3–6 m. During the day, the tentacles remain contracted and the pseudotentacles fully expanded allowing the zooxanthellae (particularly abundant in this area) to capture sunlight; at night the situation is the opposite, allowing tentacles to capture food (Sebens and DeRiemer 1977).

###### Distribution.

Western Atlantic, from Bahamas to Brazil, and along the Caribbean Sea (Wilson 1890, Manjarrés 1978, Acuña et al. 2013, Varela 2002, Herrera-Moreno and Betancourt 2002). *Lebrunia coralligens* has been recorded in the Mexican Caribbean (Jordán-Dahlgren 2008), and in the VRS (González-Muñoz 2005, see Table 1).

###### Remarks.

Currently there are two valid species of *Lebrunia*, both of them distributed in the Western Atlantic (Fautin 2013). They differ in the branched pseudotentacles: those of *Lebrunia danae* are long and dark-brown whereas those of *Lebrunia coralligens* are shorter, bright bluish-gray, with rounded ends (González-Muñoz et al. 2012).

#### Family Capneidae Gosse, 1860
Genus *Actinoporus* Duchassaing, 1850

##### Actinoporus elegans

Duchassaing, 1850

http://species-id.net/wiki/Actinoporus_elegans

Figure 8 , Table 2

Actinoporus elegans Duchassaing 1850: 10.
Actinoporus Elegans [sic]: Duchassaing 1870: 21.
Aureliana elegans : Andres 1883: 289.

###### Material examined.

La Gallega reef (19°13'20"N, 96°07'39"W; one specimen).

###### Diagnosis.

**Figure 8.**
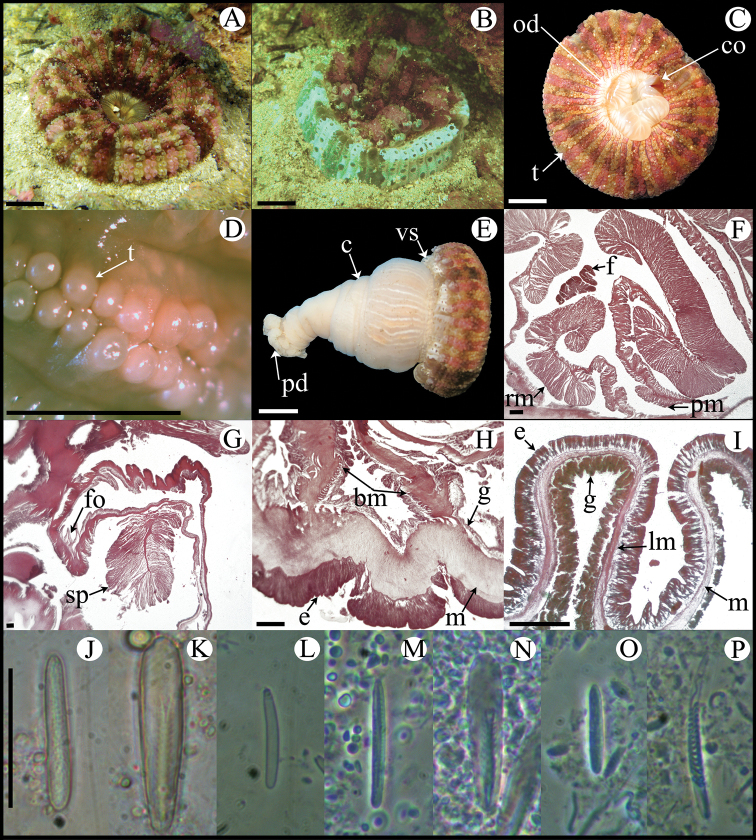
*Actinoporus elegans*. **A** Fully expanded specimen in natural habitat **B** Partially contracted specimen in natural habitat **C** Oral view **D** Detail of double rows of tentacles **E** Lateral view **F** Cross section through proximal column, showing retractor and parietobasilar muscles **G** Longitudinal section through column margin showing marginal sphincter muscle **H** Longitudinal section through base showing basilar muscles **I** Longitudinal section through tentacles **J–P** Cnidae.– actinopharynx: **J** basitrich **K** microbasic *p*-mastigophore; column: **L** basitrich; filament: **M** basitrich **N** microbasic *p*-mastigophore; tentacle: **O** basitrich **P** spirocyst. Abbreviations.– bm: basilar muscle, e: epidermis, f: filament, fo: fosse, g: gastrodermis, lm: longitudinal muscle, m: mesoglea, od: oral disc, pd: pedal disc, pm: parietobasilar muscle, rm: retractor muscle, sp: sphincter, t: tentacle, vs: vesicles. Scale bars: **A–C, E**: 10 mm; **D**: 2 mm; **F–I**: 200 μm; **J–P**: 25 μm.

Fully expanded oral disc and tentacles up to 52 mm in diameter. Central part of oral disc smooth, narrow, to 16 mm diameter, beige; mouth oval with a well-developed conchula (Figure 8C). Tentacles small, vesicle-like, arranged in double radial rows covering almost entire oral disc, on endocoelic and exocoelic spaces, 24–26 tentacles per double row; reddish or pinkish rows of tentacles alternating with pale brown rows (Figure 8A–D). Deep fosse (Figure 8G). Column elongated, funnel-shaped, to 60 mm in height, wider distally than proximally; column diameter: distally 38 mm, mid-column 27 mm, proximally 13 mm (Figure 8E). Column with longitudinal rows of vesicles (6–8 vesicles per row) distally (Figure 8B, E). Pedal disc well-developed, narrow, 19 mm in diameter. Column and pedal disc white to pale-brown; mesenterial insertions visible distally (Figure 8E). Mesenteries irregularly arranged in three cycles (28 pairs in specimen examined): first cycle perfect, others imperfect. Gametogenic tissue not observed in specimen examined. Two pairs of directives, only one pair attached to a single well-developed siphonoglyph. Retractor muscles strong, circumscribed, with main muscle lamella divided in two parts; parietobasilar muscles strong with thick mesogleal pennon (Figure 8F). Basilar muscles well-developed (Figure 8H). Marginal sphincter muscle endodermal, strong and circumscribed, pinnate (Figure 8G). Longitudinal muscles of the tentacles ectodermal (Figure 8I). Zooxanthellae absent. Cnidom: basitrichs, microbasic *p*-mastigophores, and spirocysts (Figure 8J–P, Table 2).

###### Natural history.

*Actinoporus elegans* inhabits sandy bottoms, at 1–2 m; the column is burrowed in the sand but the pedal disc is strongly attached to rocks. When disturbed, it contracts the oral disc suddenly, completely burrowing the entire body.

###### Distribution.

Western Atlantic, from the northern coast of Brazil to Guadeloupe, Jamaica, and Curaçao (Corrêa 1973), and Cape Verde Islands (Wirtz 2009). This is the first record for the coast of Mexico; found in the VRS.

###### Remarks.

Currently there are two valid species of *Actinoporus*: *Actinoporus elegans* and *Actinoporus elongatus* Carlgren, 1900 (Fautin 2013). *Actinoporus elongatus* is reported for India, Mozambique and Australia (Carlgren 1900, Menon 1927, Clayton and Collins 1992), and it lacks the longitudinal rows of vesicles in the distal column of *Actinoporus elegans* (Carlgren 1900, Corrêa 1973). Additional color patterns observed for *Actinoporus elegans* in coral reefs off the coast of Venezuela include tentacles and oral disc almost completely white with dark-brown stripes, or completely bright orange (unpublished data).

#### Superfamily Metridioidea Carlgren, 1893
Family Hormathiidae Carlgren, 1932
Genus *Calliactis* Verrill, 1869

##### Calliactis tricolor

(Le Sueur, 1817)

http://species-id.net/wiki/Calliactis_tricolor

Figure 9 , Table 2

Actinia tricolor Le Sueur 1817: 171.
Actinia bicolor Le Sueur 1817: 171.
Cereus bicolor : Milne-Edwards 1857: 273.
Adamsia tricolor : Milne-Edwards 1857: 281.
Adamsia Egletes [sic] Duchassaing and Michelotti 1864: 40.
Adamsia egletes : Duchassaing and Michelotti 1866: 134.
Calliactis bicolor : Verrill 1869: 481.
Adamsia sol McMurrich 1893: 183.
Adamsia bicolor : Andres 1883: 179.
Adamsia tricolor Andres 1883: 180.
Calliactis tricolor : Haddon 1898: 457.

###### Material examined.

Alacranes reef (22°31'35"N, 89°46'05"W; eight specimens), Serpientes reef (21°26'22"N, 90°28'25"W; five specimens).

###### Diagnosis.

**Figure 9.**
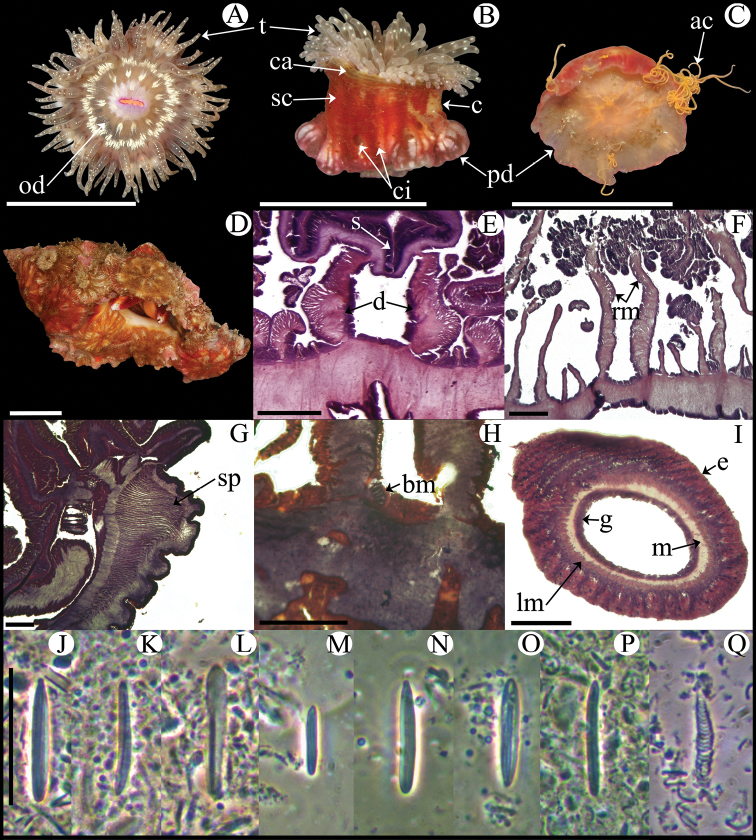
*Calliactis tricolor*. **A** Oral view **B** Lateral view **C** Pedal disc view **D** Specimens on hermit crab shell **E** Detail of directives showing a siphonoglyph **F** Cross section through proximal column **G** Longitudinal section through margin showing the marginal sphincter muscle **H** Longitudinal section through base showing basilar muscles **I** Cross section through tentacle **J–Q** Cnidae.– acontio: **J** basitrich; actinopharynx: **K** basitrich **L** microbasic *p*-mastigophore; column: **M** small basitrich; filament: **N** basitrich **O** microbasic *p*-mastigophore; tentacle: **P** basitrich **Q** spirocysts. Abbreviations.– ac: acontia, bm: basilar muscle, c: column, ca: capitulum, ci: cinclides, e: epidermis, g: gastrodermis, lm: longitudinal muscle, m: mesoglea, od: oral disc, pd: pedal disc, rm: retractor muscle, sc: scapus, sp: sphincter, t: tentacle. Scale bars: **A–D**: 10 mm; **E–I**: 200 μm; **J–Q**: 25 μm.

Fully expanded oral disc and tentacles 9–48 mm in diameter. Oral disc smooth, wider than column, 3–20 mm in diameter, pale-brown translucent, with small white stripes in endocoelic spaces, sometimes forming a white ring; some specimens also with pink flashes (Figure 9A). Mouth bright yellow, orange, or white; often with purple ring around lips (Figure 9A). Tentacles hexamerously arranged in 5–6 cycles (96–192 in number), smooth, thin, short (2.5–15.5 mm), inner ones longer than outer ones, contractile (Figure 9A, B), tapering distally, pale-brown with a longitudinal row of white dots along entire length (Figure 9A, B); some specimens also with bright-pink flashes mainly at tips. Column trumpet-shaped in extended position, dome-shaped when contracted, 5–24.5 mm in diameter and 4–31 mm in height, divided into narrow, smooth capitulum and wrinkled-texture scapus (Figure 9B). Capitulum pale-brown to yellowish, scapus bright to dark orange often with small white stripes or white flashes slightly above limbus (Figure 9B). Pedal disc well-developed, circular to irregular, wider than column, 6–36 mm in diameter, with mesenterial insertions visible, pale-brown and translucent (Figure 9C). One or two rows of cinclides proximally, near limbus; dark-red or brown (Figure 9B). Mesenteries hexamerously arranged in four cycles; same number of mesenteries proximally and distally (to 48 pairs in specimens examined): first cycle perfect, others imperfect; third and fourth cycles poorly developed, without filaments or acontia. Gametogenic tissue not observed in specimens examined. Two pairs of directives each attached to a well-developed siphonoglyph (Figure 9E). Retractor muscles weak and diffuse; parietobasilar muscles poorly developed (Figure 9E, F). Basilar muscles poorly developed (Figure 9H). Marginal sphincter muscle mesogleal, strong, transversally stratified (Figure 9G). Longitudinal muscles of tentacles ectodermal. Acontia numerous, bright orange (Figure 9C), with basitrichs. Zooxanthellae present. Cnidom: basitrichs, microbasic *p*-mastigophores, and spirocysts (Figure 9J–Q; see Table 2).

###### Natural history.

*Calliactis tricolor* typically dwells on the shells of living hermit crabs often carrying more than one individual (Figure 9D), between 10–30 m. This peculiar symbiotic relationship has been widely studied (reviewed in Gusmão and Daly 2010).

###### Distribution.

Western Atlantic, from the northern coast of USA to the northern coast of Brazil, along the Caribbean Sea and Gulf of Mexico (Carlgren and Hedgpeth 1952, Zamponi et al. 1998). This is the first record for the coast of Mexico; found in Serpientes and Alacranes reefs.

###### Remarks.

Of the 18 valid species currently considered as valid of *Calliactis*, only two have been reported in the Gulf of Mexico and Caribbean Sea (Fautin 2013): *Calliactis polypus* (Forsskål, 1775) and *Calliactis tricolor*. These two species differ in the color of the cinclides, white in *Calliactis polypus* and dark-red in *Calliactis tricolor* (Gusmão 2010). In addition, *Calliactis tricolor* is distributed almost exclusively in the western Atlantic whereas *Calliactis polypus* has a wide distribution range, being found in the Red Sea, Hawaii, French Polynesia, Australia, South Africa, East Africa, Maldives, Cape Verde Islands, Japan, Galapagos, and Louisiana (Gusmão 2010, Fautin 2013).

## Supplementary Material

XML Treatment for Anemonia sargassensis

XML Treatment for Anthopleura pallida

XML Treatment for Bunodosoma cavernatum

XML Treatment for Isoaulactinia stelloides

XML Treatment for Lebrunia coralligens

XML Treatment for Actinoporus elegans

XML Treatment for Calliactis tricolor
